# Proteomics and EPS Compositional Analysis Reveals *Desulfovibrio bisertensis* SY-1 Induced Corrosion on Q235 Steel by Biofilm Formation

**DOI:** 10.3390/ma17205060

**Published:** 2024-10-17

**Authors:** Yanan Wang, Ruiyong Zhang, Krishnamurthy Mathivanan, Yimeng Zhang, Luhua Yang, Fang Guan, Jizhou Duan

**Affiliations:** 1 Key Laboratory of Advanced Marine Materials, Key Laboratory of Marine Environmental Corrosion and Bio-Fouling, Institute of Oceanology, Chinese Academy of Sciences, Qingdao 266071, China; wangyanan@qdio.ac.cn (Y.W.); kritamathi@qdio.ac.cn (K.M.); zhangyimeng@qdio.ac.cn (Y.Z.); yangluhua@qdio.ac.cn (L.Y.); guanfang@qdio.ac.cn (F.G.); 2 University of Chinese Academy of Sciences, Beijing 100049, China; 3 Guangxi Key Laboratory of Marine Environmental Science, Institute of Marine Corrosion Protection, Guangxi Academy of Sciences, Nanning 530007, China

**Keywords:** planktonic cells, biofilm formation, microbially influenced corrosion, extracellular polymeric substances, proteomics

## Abstract

Microorganisms that exist in the seawater form microbial biofilms on materials used in marine construction, especially on metal surfaces submerged in seawater, where they form biofilms and cause severe corrosion. Biofilms are mainly composed of bacteria and their secreted polymeric substances. In order to understand how biofilms promote metal corrosion, planktonic and biofilm cells of *Desulfovibrio bizertensis* SY-1 (*D. bizertensis*) from Q235 steel were collected and analyzed as to their intracellular proteome and extracellular polymeric substances (EPS). The intracellular proteome analysis showed that the cellular proteins were strongly regulated in biofilm cells compared to planktonic cells, e.g., along with flagellar proteins, signaling-related proteins were significantly increased, whereas energy production and conversion proteins and DNA replication proteins were significantly regulated. The up-and-down regulation of proteins revealed that biofilm formation by bacteria on metal surfaces is affected by flagellar and signaling proteins. A significant decrease in DNA replication proteins indicated that DNA is no longer replicated and transcribed in mature biofilms, thus reducing energy consumption. Quantitative analysis and lectin staining of the biofilm on the metal’s surface revealed that the bacteria secreted a substantial amount of EPS when they began to attach to the surface, and proteins dominated the main components of EPS. Further, the infrared analysis showed that the secondary structure of the proteins in the EPS of the biofilm was mainly dominated by β-sheet and 3-turn helix, which may help to enhance the adhesion of EPS. The functional groups of EPS analyzed using XPS showed that the C element of EPS in the biofilm mainly existed in the form of combinations with N. Furthermore, the hydroxyl structure in the EPS extracted from the biofilm had a stronger hydrogen bonding effect, which could maintain the stability of the EPS structure and biofilm. The study results revealed that *D. bizertensis* regulates the metabolic pathways and their secreted EPS structure to affect biofilm formation and cause metal corrosion, which has a certain reference significance for the study of the microbially influenced corrosion (MIC) mechanism.

## 1. Introduction

Metallic materials are widely used in ships, pipelines, marine engineering, etc., and undergo severe corrosion due to the special characteristics of the marine environment. The main microorganisms present in the marine environment play an important role in the process of metal corrosion. The formation of biofilm by microorganisms on metal surfaces is the initial stage of microbial corrosion, as evidenced by research [1,2,3,4]. The biofilm structure provides microorganisms with a unique living space that allows them to remain viable in harsh conditions. The close contact between the biofilm and the metal surface creates an absolute anaerobic environment within the biofilm, which promotes the growth of anaerobic bacteria such as sulfate reducing bacteria (SRB) [5].

SRB are the main microorganisms that cause metal corrosion, and they are widely found in marine structures and pipelines [6,7,8]. In particular, mature SRB biofilm, during their growth and metabolism, produce corrosive metabolites such as organic acids, sulfides, etc., which cause chemical corrosion on metals [9,10,11]. More and more studies have proved that the corrosion of carbon steel by SRB is associated with extracellular electron transfer; SRB can directly utilize metallic materials such as carbon steel as electron donors to reduce the sulfate in their cytoplasm [12,13]. MIC directly caused by anaerobic biofilms is classified into two main types based on their mechanisms: extracellular electron transfer MIC (EET-MIC) and metabolite MIC (M-MIC) [9,14,15,16]. The electron transfer pathway of mature SRB biofilms on the surface of carbon steel is even more significant, resulting in electrochemical microbial corrosion that accelerates the corrosion of metals [17]. It has also been determined that SRB in sea mud have established significant biofilms on sand grains and transformed the sea mud into an electronic conductor [18].

Biofilms are composed of microorganisms and their secreted polymers (EPS), which support microbial survival amidst harsh conditions and promote the adhesion and aggregation of microbial cells [19,20]. It has been reported that SRB biofilms are composed of proteins, along with small amounts of extracellular polysaccharides [21,22]. The biofilms of SRB have been extensively studied over the past few decades, and the differentials of genes and proteins between biofilm cells and planktonic cells have been explored [23]. Physiological differences between biofilm cells and planktonic cells are due to alterations in genes/proteins related to carbon flow and extracellular structures. Biofilm cells alter the abundance of transcripts and proteins involved in carbon and energy metabolism, amino acid metabolism, stress response, proteases, and ribosomal proteins [24,25]. Besides *Desulfovibrio vulgaris* and *Desulfovibrio alaskensis*, few other genes and proteins of SRB have been studied at the molecular level, and the mechanism by which SRB biofilms contribute to metal corrosion has not been determined.

Biofilm cells have a lifestyle distinct from those of planktonic cells, and both types of cells can be regulated by a population sensing system [26]. This can be interpreted at the molecular level by tools such as proteomics. The application of proteomics can help to understand the metabolic activity of bacteria during corrosion. EPS, as a matrix for the protection and communication of biofilm cells, is considered a key component in determining the physical, chemical, and biological properties of biofilms [20]. Some specific functions of biofilms depend on EPS, and therefore analysis of EPS can be effective in understanding the regulatory mechanisms of biofilms and the mechanisms of corrosion. To date, although the biological and electrochemical mechanisms of SRB in sulfate-rich environments have been extensively studied, there is a lack of research on the molecular regulatory mechanisms of SRB biofilms and the relationship between biofilms and corrosion. Therefore, this study aimed to elucidate the mechanism of metal corrosion caused by SRB biofilm, using proteomics and EPS analysis.

## 2. Materials and Methods

### 2.1. Microorganism, Medium, and Cultivation

*Desulfovibrio bizertensis* SY-1 (NCBI: PRJNA790473), a sulfate-reducing bacterium used in this experiment, was isolated and purified from a rusted layer of carbon steel which had been immersed in the South China Sea for a long time. For cultivation, the growth medium modified Postgate C (PGC) medium was used. The modified Postgate C (PGC) medium contained 0.5 g KH_2_PO_4_, 1 g NH_4_Cl, 0.06 g CaCl_2_-6H_2_O, 0.06 g MgSO_4_-7H_2_O, 6 mL 70% sodium lactate, 1 g yeast extract, and 0.3 g sodium citrate in 1 L of aged seawater from the Qingdao offshore area. Before sterilization, the PGC was purged with N_2_ for at least 45 min. The steel used in the experiments was Q235 mild steel, with dimensions of 80 mm × 30 mm × 3 mm, and its chemical composition (mass fraction) was: C 0.14%, Mn 0.30%, Si 0.30%, S 0.050%, P 0.045%, with the rest of the composition being Fe. Before the experiment, it was polished with 1500# and 2000# water sandpaper, dehydrated by anhydrous ethanol, ultrasonication was performed for 10 min, and the sample was vacuum dried, weighed, and set aside.

Corrosion experiments were conducted by adding three Q235 steel sheets to 500 mL jars containing 300 mL of PGC medium. Prior to bacterial inoculation, the jars were corked with a rubber stopper and sealed with epoxy resin. Then, 3 mL (1% *v*/*v*) of *D. bizertensis* (bacterial count of 1 × 10^5^) was injected into the jars using a sterile syringe and then incubated at 30 °C for 14 days. All of the experiments were performed in the anaerobic chamber.

### 2.2. EPS Extraction, Quantification, Analysis, Staining, and Observation

EPS of planktonic and biofilm cells of *D. bizertensis* were extracted using the cation exchange resin (CER). The biofilm of *D. bizertensis* was scraped from a Q235 steel sheet using a sterile scalpel blade, suspended in sterile 0.9% NaCl solution, and centrifuged at 5000× *g* for 10 min at 4 °C to collect the cells. The collected cell precipitate was washed twice with sterile 0.9% NaCl, resuspended in sterile 0.9% NaCl (30 mL), and added to 70 g resin/g VSS cation exchange resin (DOWEX 50 × 8, Na^+^ form, 20–50 mesh, Sigma-Aldrich, St. Louis, MO, USA); it was then extracted at 120 rpm and 4 °C for 6 h. Then, the suspension was centrifuged (10,000× *g*, 15 min, 4 °C) to collect the supernatant. The EPS was collected from the supernatant by filtration using a 0.45 μm membrane filter and the extracted EPS was dialyzed at 4 °C using a 3.5 KDA dialysis bag. For efficient dialysis, the dialysis solution (ultrapure water) was changed every 4 h. The dialyzed EPS solution was freeze-dried using a freeze dryer for 72 h. The cell precipitate after EPS extraction was observed to check cell lysis, using SEM and fluorescence microscope (FM) (OLYMPUS DP80, Tokyo, Japan).

X-ray photoelectron spectroscopy (XPS, ESCALAB 250XI, Thermo Fisher, Waltham, MA, USA) and Fourier transform infrared spectroscopy (FTIR, Thermo Nicolet Is50, Waltham, MA, USA) were used to determine the chemical composition of EPS, such as element valence and functional groups, respectively. For FTIR analysis, the EPS samples were ground and molded into discs using IR-grade KBr powder. To determine the secondary structure of the protein, infrared analysis was performed using Peakfit V4 software. The second-order derivatives of the amide I region at 1700–1600 cm^−1^ were generated and smoothed by 15% [27]. A Gaussian line shape was selected for quantitative analysis and the fitting process was performed with a correlation (r^2^) ≥ 0.999 in all of the fitted spectra.

The planktonic cells were removed by rinsing the Q235 steel surface containing biofilm using sterile phosphate-buffered saline (PBS, pH = 7.4). The polysaccharides of biofilm on Q235 steel were determined by staining using tetramethyl rhodamine isothiocyanate-conjugated concanavalin A (ConA-TRITC, 0.1 mg/mL) and kept for 20 min in a dark room. Then, the Q235 steel was stained with fluorescein isothiocyanate (FITC) protein stain (20 μL/mL) to detect the proteins and stained with Nile red solution (10 mg/L, 60 μL) for 10 min to detect the lipids on the biofilm. A fluorescence microscope (FM) (OLYMPUS DP80, Tokyo, Japan) was used to investigate the distribution of polysaccharides and proteins in the biofilm EPS on the Q235. The FM was adjusted to 557/580 nm for ConA-TRITC, 514/625–700 for the Nile red, and 495/525 nm for the FITC.

The planktonic and biofilm cells were counted using plate counting. The proteins, polysaccharides, and DNA present in the biofilm EPS were measured according to the Lowry assay [28], the anthrone method [29], and the diphenylamine colorimetric method [30], respectively.

### 2.3. Biofilm Observation on the Q235 Surface

The live/dead cells in biofilm on the Q235 surface were observed by a laser scanning confocal microscope (LMS900, Carl Zeiss, Oberkochen, Germany) using LIVE/DEAD Viability kit (SYTO™ 9 and Propdium iodide, Invitrogen, Thermo Fisher Scientific, Waltham, MA, USA) as a fluorescent stain. The rust layer and biofilm morphology on the surface of Q235 were observed using scanning electron microscopy (SEM, FEI Quanta250, Thermo Fisher, Waltham, MA, USA), with 5–20 kV accelerating voltage. For SEM analysis, the Q235 samples were collected from the bacterial solution and placed in 2.5% (*v*/*v*) glutaraldehyde solution for 4 h. Then, the samples were dehydrated using various ethanol concentrations (25%, 50%, 60%, 70%, 80%, 90%, and 100%) and subjected to SEM analysis.

The crystal structure of the rust layer of Q235 steel was investigated by XRD (Rigaku D/max-Ultima IV, Tokyo, Japan) in the range of 10–90° at 5°/min under Cu Ka radiation (=0.1542 nm), 40 kV, 30 mA. After 14 days, the Q235 was collected from the medium containing *D. bizertensis* and cleaned with a carbon steel descaling solution according to the national standard [31] (ISO 8501:2007, IDT) to completely remove the corrosion products, then washed with deionized water and ethanol, and finally dried with N_2_ and weighed. The rate in mm/a was calculated from the specific weight loss obtained from the following equation:

V_corr_ = (87600 × ∆m)/ρAt (1)

where V_corr_, ∆m, ρ, A, and t are the coupon corrosion rate (mm/a), weight loss (g), coupon density (g·cm^−3^), exposed coupon area (cm^2^), and incubation time (h), respectively.

To determine the morphology and depth of corrosion pits, the Q235 steel samples were analyzed using a confocal laser scanning microscope (OLYMPUS CLSM, Lext OLS5000, Tokyo, Japan) after the corrosion products were removed.

### 2.4. Proteome Analysis

The details of the proteome analysis are described in the Supplementary Materials. Firstly, the total protein of *D. bizertensis* was extracted, quantified, detected, digested, and desalted. Secondly, TMT (Tandem mass tags) Labeling of Peptides was performed. Thirdly, the sample was fractionated using a C18 column (Waters BEH C18, 4.6 mm × 250 mm, 5 μm) on a Rigol L3000 HPLC system (Thermo Fisher, Waltham, MA, USA). Fourth, an EASY-nLCTM 1200 UHPLC system (Thermo Fisher, Waltham, MA, USA) was coupled with a Q ExactiveTM HF-X mass spectrometer (Thermo Fisher, Waltham, MA, USA) operating in the data-dependent acquisition (DDA) mode for LC-MS/MS analysis. Finally, the identification, quantitation, and function analysis of the protein and differentially expressed proteins (DEPs) were performed. To increase the reliability of the measurements, at least three parallel assays were applied, and the results were presented as the average of the experiments.

## 3. Results

### 3.1. Biofilm Morphology and Growth, and the Composition Analysis of D. bizertensis on the Q235 Surface

**Figure 1:**
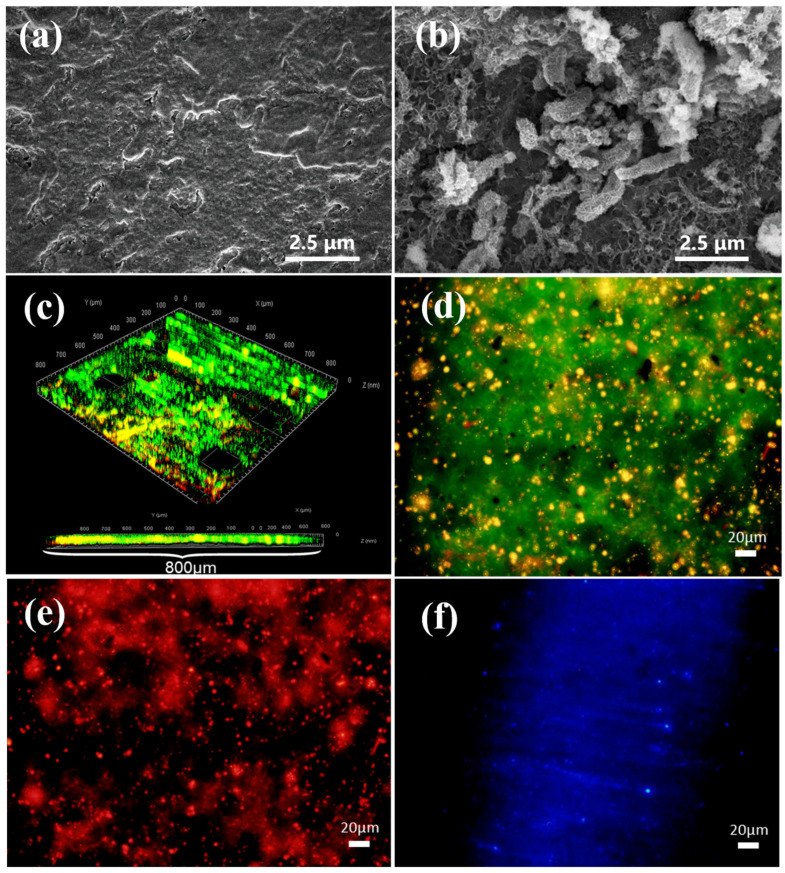
Scanning electron microscopy (SEM) micrograph images of the Q235 surface from (**a**) control and (**b**) *D. bizertensis*-containing medium. Confocal laser scanning microscopy images of the biofilm cells on the Q235 surface after 15 days immersion, stained with the LIVE/DEAD Biofilm Viability kit (**c**), and EPS distribution ((**d**) proteins stained with FITC, (**e**) polysaccharides stained with Concanavalin A-TRITC, and (**f**) lipids stained with Nile red). Green coloring indicates live cells, red coloring indicates dead cells, and yellow coloring indicates partially damaged/dead cells.

**Figure 2:**
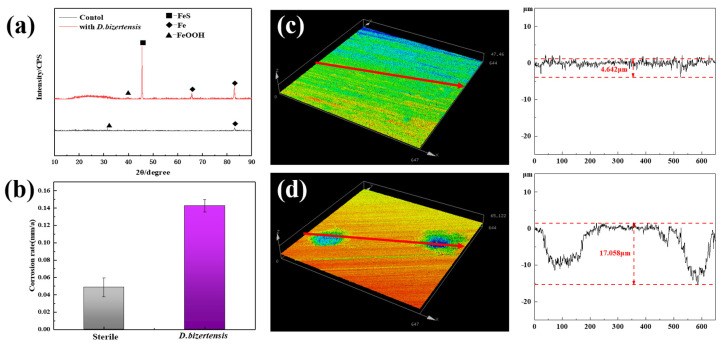
(**a**) XRD of surface products of Q235 steel from control and *D. bizertensis*-containing medium, and (**b**) annual corrosion rates of Q235 steel after 15 days incubation. (**c**,**d**) The pits and their depths on the Q235 steel coupons from control and *D. bizertensis*-inoculated media after 15 days.

The SEM image shows the cellular state of *D. bizertensis* on the surface of Q235 (Figure 1a,b). It can be seen that *D. bizertensis* secreted abundant flocculent material around the bacteria and that this flocculent material appeared in a honeycomb-like shape (Figure 1a,b). The confocal laser scanning microscopy image of the biofilm cells stained with the LIVE/DEAD Biofilm Viability kit is shown in Figure 1c. The CLSM image showed that live microorganisms were dominant on the metal surface at 14 days (Figure 1c). The composition of the EPS of biofilm on the Q235 surface stained with fluorescence dye is shown in Figure 1d–f; it shows that the EPS of biofilm on the Q235 surface consisted mostly of proteins and polysaccharides, as well as a small volume of lipids. Further, the fluorescence signal intensity of the protein was higher than those of the other components, indicating that the bacteria may secrete large volumes of proteins for biofilm formation. The weight loss studies revealed that the corrosion rate of Q235 steel reached 0.142 mm/a in the medium containing *D. bizertensis* (Figure 2a). The results of XRD revealed that the corrosion products on the Q235 surface mainly contained FeS (Figure 2b). CLSM images showed many pitting pits on the Q235 surface after removal of the corrosion products from the Q235 surface, and the depth of maximum pitting pits was 17.058 μm (Figure 2c,d). These findings indicated that *D. bizertensis* caused corrosion on the Q235 steel surface, mainly pitting corrosion.

### 3.2. Identification and Comparison of Proteins

**Figure 3:**
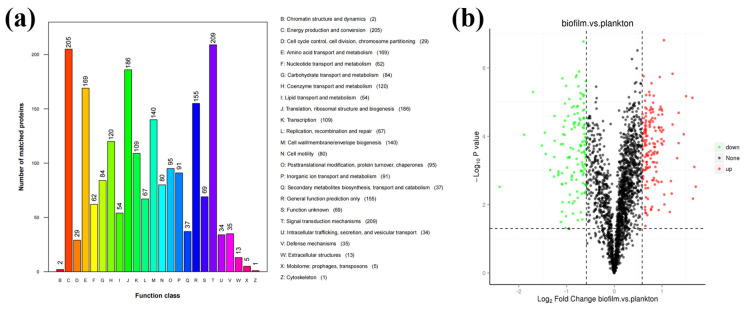
(**a**) Summary of detectable protein contents and functions. (**b**) Volcano plots representing the results of the proteome analysis in biofilm vs. planktonic cells.

*D. bizertensis* biofilm and its relationship to metal corrosion were studied by analyzing the changes in their metabolic pathways using proteomic analysis. Statistical analysis of the obtained protein samples showed that most of the proteins were associated with signal transduction mechanisms (number of matching proteins: 209), energy production and conversion (number of matching proteins: 205), translation, ribosome structure and biogenesis (number of matching proteins: 186), amino acid translocation and metabolism (number of matching of proteins: 169), and cell wall/membrane/envelope biogenesis (number of matching proteins: 140) (Figure 3a,b). The differences in protein expression between biofilm and planktonic cells of *D. bizertensis* after 7 days of incubation, detected from volcano plots, are shown in Figure 3b. Differences between proteins were referred to as differentially expressed proteins (DEPs). A total of 154 up-regulated proteins and 130 down-regulated proteins were found. All differentially expressed proteins determined to be up-regulated (FC ≥ 1.5 while *p*-value ≤ 0.05) or down-regulated (FC ≤ 0.67 while *p*-value ≤ 0.05) are shown in Tables S1 and S2.

**Figure 4:**
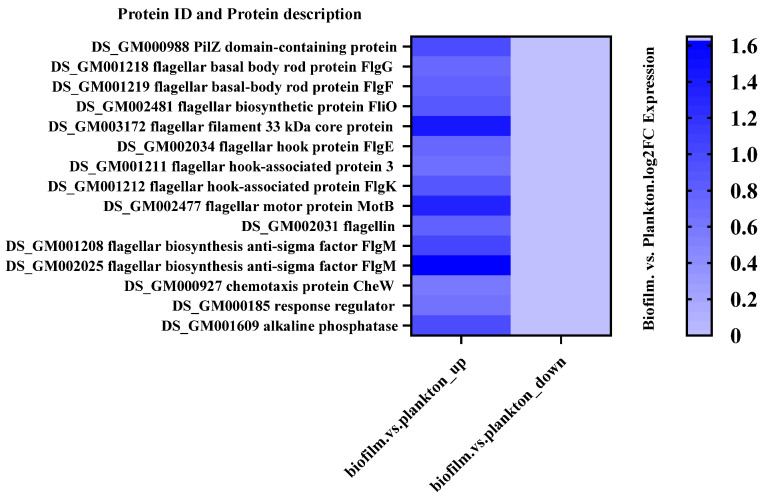
Up- and down-regulated flagellum-related DEPs in biofilm and planktonic cells.

A number of DEPs were selected to demonstrate significant differences in protein expression between biofilm cells and planktonic cells. The results (Figure 4 and Tables S1 and S2) showed that the proteins related to flagellum and signaling were up-regulated and proteins related to energy production and conversion as well as proteins related to translation, ribosomal structure, and biogenesis were down-regulated in biofilm cells compared to plankton. Figure S1 depicts the subcellular locations of DEPs, revealing that 52.38% of DEPs were found in the cytoplasm, 38.1% in the cellular membrane, and the remainder extracellularly. This indicated that changes in the cytoplasm and the membrane of *D. bizertensis* were responsible for corrosion on Q235 carbon steel.

The biological functions affected by DEPs were categorized into biological process, molecular function, and cellular component using the GO analysis tool and analyzed to compare the biological functions affected in biofilm cells and planktonic cells on metal surfaces. The GO analysis (Table S3) showed that DEPs were significantly enriched in biological process (BP), cellular component (CC), and molecular function (MF). Further, most DEPs in BP were determined to be significantly enriched in cellular process (GO:0009999) and molecular function (MF). Cellular component (CC) and molecular function (MF) were significantly enriched due to the presence of a large number of DEPs in BP, with significant enrichment of cellular process (GO:0009987), biosynthetic process (GO:0009058), cellular nitrogen compound metabolic process (GO:0034641), cellular biosynthetic process (GO:0044249), and organic substance biosynthetic process (GO:1901576), which were enriched with 60, 28, 27, 27, and 27 DEPs, respectively. In the cellular component (CC), the cell part (GO:0044464), membrane (GO:0016020), and cytoplasm (GO:0005737) were enriched with 22, 20, and 17 DEPs, respectively. In MF, the structural molecule activity (GO:0005198), structural constituent of ribosome (GO:0003735), ion binding (GO:0043167), and metal ion binding (GO:0046872) were enriched in 17, 14, 12, and 11 DEPs, respectively. This indicated that metal surface biofilm and planktonic DEPs are mainly enriched in the biosynthetic and metabolic processes of BP, the cellular part of CC, the cell membrane and cytoplasm, the structural molecular activities of MF, and the binding of ribosomes and ions.

**Figure 5:**
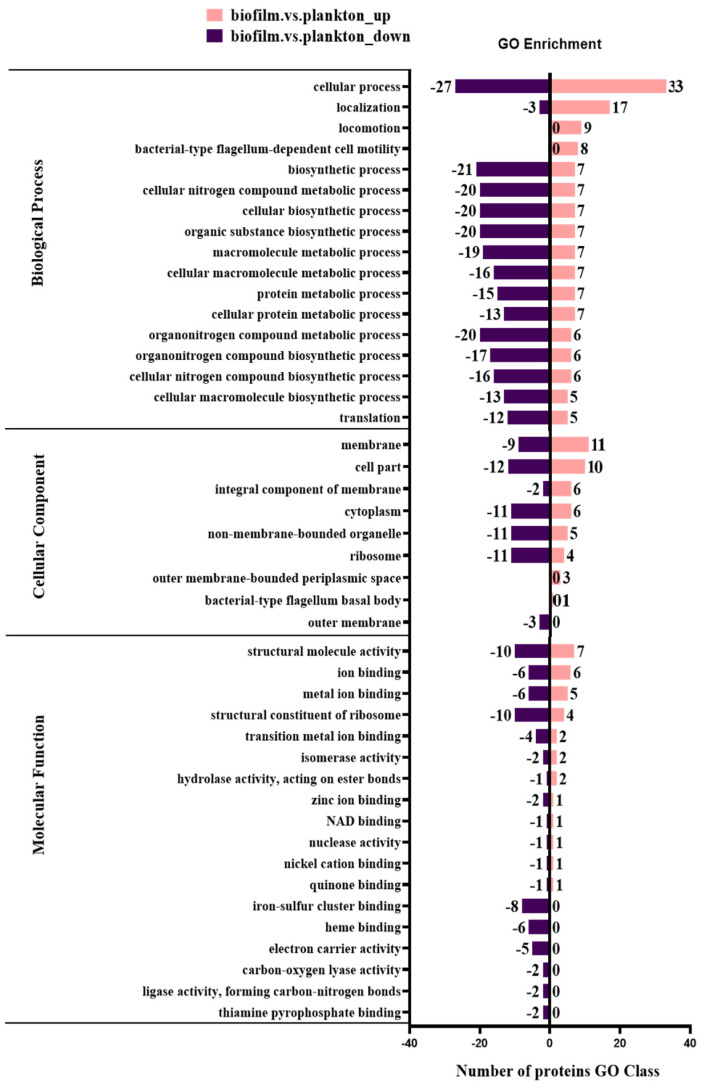
Functional category distribution of differentially expressed proteins by GO in biofilm and planktonic cells.

The functional differences between up-regulated and down-regulated proteins were analyzed using the GO terms (BP/CC/MF) of quantified proteins (Figure 5). A total of 154 proteins were up-regulated in biofilm cells on the Q235 surface, and they were enriched in cellular process (GO:0009987), localization (GO:0051179), membrane (GO:0016020), locomotion (GO:0040011), and bacterial-type flagellum-dependent cell motility (GO:0071973). Also, the 130 proteins were down-regulated in biofilm cells and they were enriched in the biosynthetic process (GO:0009058), cellular nitrogen compound metabolic process (GO:0034641), protein metabolic process (GO:0019538), iron–sulfur cluster binding (GO:0051536), heme binding (GO:0020037), and electron carrier activity (GO:0009055).

**Figure 6:**
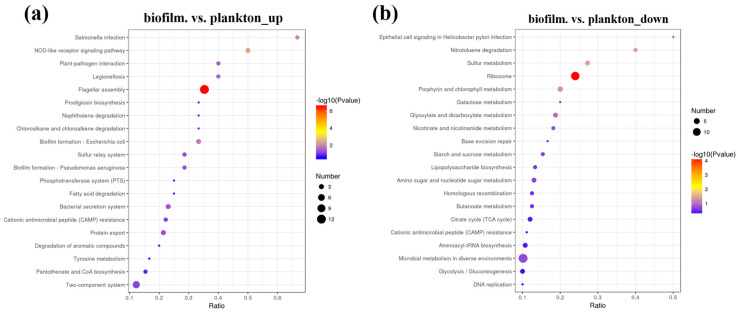
Differentially expressed proteins in KEEG pathway biofilm cells on the Q235 surface and planktonic cells, (**a**) biofilm.vs. plankton_up, (**b**) biofilm.vs.plankton_down.

Biological functions in organisms are activated by interactions between various proteins, and the significant enrichment of the KEEG pathway can identify the most important metabolic pathways and signaling pathways involving DEPs. Figure 6, Tables S4 and S5 shows the DEPs in KEEG pathways of biofilm cells on the Q235 surface and planktonic cell. The significant enrichment in DEPs of the KEEG pathway was mainly in the ribosome and flagellar assembly. The proteins up-regulated in biofilm cells significantly enriched the flagellar assembly (map02040), two-component system (map02020), ribosome (map03010), ABC transporters (map02010), biofilm formation (map02026), bacterial secretion system (map03070), sulfur relay system (map04122), quorum sensing (map02024), bacterial chemotaxis (map02030), oxidative phosphorylation (map00190), and others. Contrastingly, down-regulated proteins in biofilm cells were significantly enriched in ribosome (map03010), carbon metabolism (map01200), porphyrin and chlorophyll metabolism (map00860), sulfur metabolism (map00920), and others.

**Figure 7:**
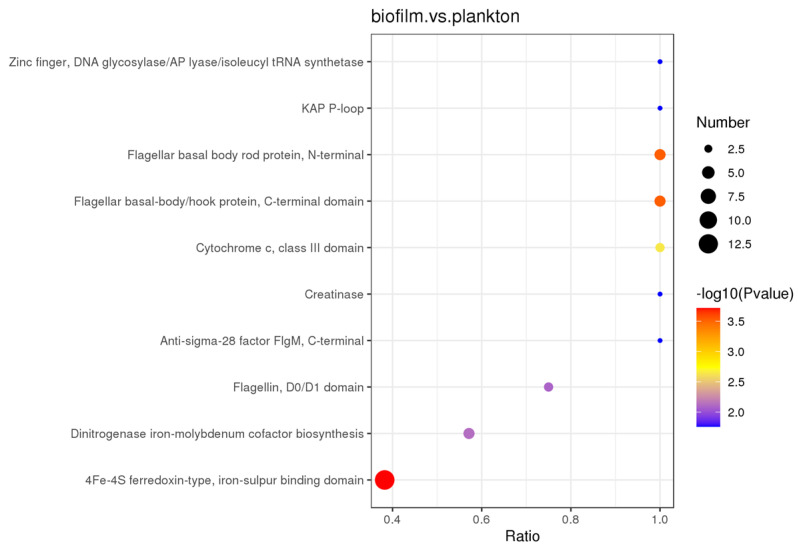
Intermittent protein restriction of differentially expressed proteins in biofilm cells on the Q235 surface and in planktonic cells.

DEPs in both biofilm and planktonic cells were also examined using protein structural domain enrichment analysis, and it was found that biofilm and planktonic DEPs were mainly enriched in 4Fe-4S ferredoxin-type, iron–sulfur binding domain, flagellar basal body rod protein, N-terminal, flagellar basal-body/hook protein, C-terminal domain, dinitrogenase iron-molybdenum cofactor biosynthesis, cytochrome c, class III domain, extracellular solute-binding protein, family 3, etc., as shown in Figure 7.

Proteomics results indicated that the DEPs have a wide range of functions, which can be categorized into cellular components, molecular functions, and biological processes. The relevant metabolic pathways were mainly related to ribosomes, flagellar assembly, sulfur metabolism, ABC transporter proteins, and energy metabolism. The relevant protein structural domains were mainly involved in 4Fe-4S ferredoxin-type, flagellar, and thioredoxin-reducing proteins, etc.

### 3.3. Changes in EPS during Biofilm Formation on the Q235 Surface

**Figure 8:**
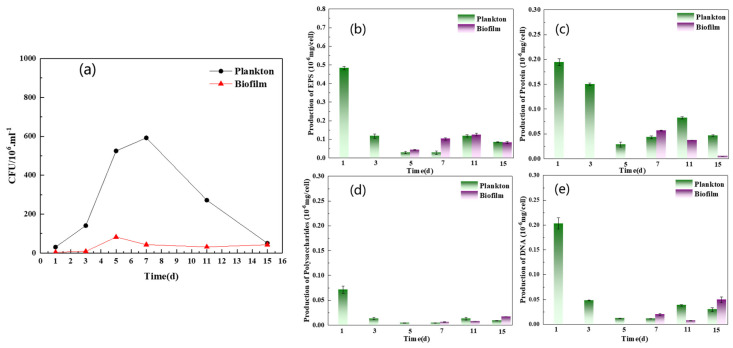
Growth curve (**a**) and EPS concentration of plankton cell and biofilm cell: (**b**) total, (**c**) protein, (**d**) polysaccharide, and (**e**) DNA.

The growth rate and EPS compositional analysis of planktonic and biofilm cells are shown in Figure 8a–d. As shown in Figure 8a, a large number of *D. bizertensis* biofilm cells were attached on the fifth day, and the number remained almost unchanged after the seventh day, indicating that the biofilm structure was relatively stable. Contrastingly, the growth of planktonic cells gradually increased and peaked on the seventh day and then decreased; this might be due to nutrient depletion in the media after the seventh day, as the planktonic cells consume nutrients from the media during their early stage of growth. However, the biofilm cells were not affected by nutrient depletion, and this might be due to the stability of the biofilm.

**Figure 9:**
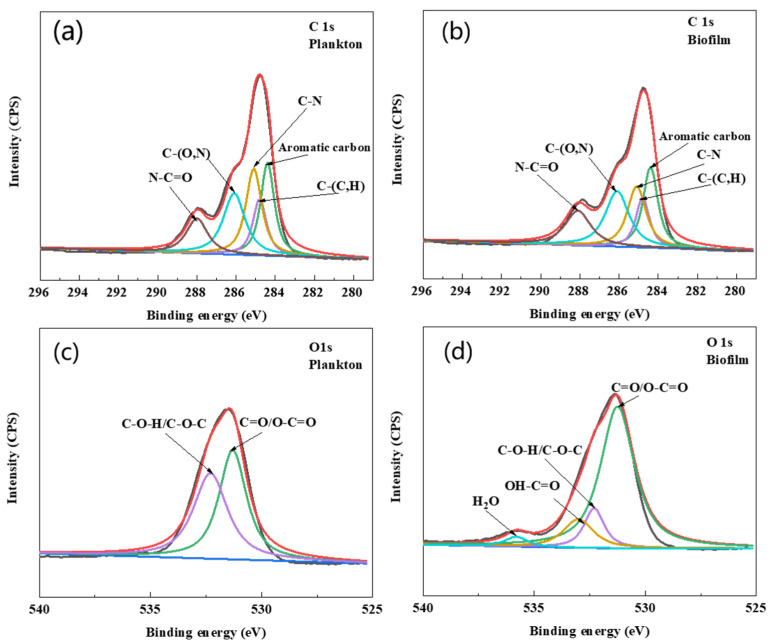
Deconvoluted C 1s and O 1s: High-resolution XPS spectra of EPS of biofilm and planktonic cells. (**a**) EPS of planktonic cells C 1s, (**b**) EPS of biofilm cells C 1s, (**c**) EPS of planktonic cells O 1s, (**d**) EPS of biofilm cells O 1s.

The EPS composition of biofilm on the surface of Q235 and planktonic cells is shown in Figure 8b–d. The results showed that the amount of EPS was greater in planktonic cells than in biofilm cells during the first two days. However, after two days, the amount of EPS was higher in biofilm cells than in planktonic cells. The compositional analysis revealed that proteins were predominant in the EPS of biofilm and planktonic cells over other components, and this was consistent with the results of agglutinin staining. Further, a higher content of DNA in the EPS of planktonic cells was observed during the early stage of growth. It has been reported that DNA can act as a signaling molecule to allow planktonic cells to aggregate and form a biofilm [32,33]. The cell morphology of planktonic and biofilm cells captured by SEM and fluorescence microscopy is shown in Figure S1. As shown in Figure S1, the cells were not damaged and remained active after EPS extraction using CER. XPS spectra (Figure 9a,b and Table 1) revealed that the C element of EPS in the biofilm was mainly combined with N (about 64%), and the C of aromatic hydrocarbon was 23%. In the EPS of planktonic cells, the C element was combined with N functional group and accounted for 62.2%, which was about 1.8% less than that in the EPS of biofilm, but the concentration of aromatic C increased by about 0.5%. The combination of C and N was related to the amide structure of the proteins, and the aromatic hydrocarbons are related to the metabolism of bacteria. The element O was C=O/O-C=O and it was mainly present in the amide I structure (Figure 9c,d and Table 1). The percentages of C=O/O-C=O in planktonic and biofilm cells were about 49.3% and 71.9%, respectively. Also, the amount of OH-C=O also corresponded to the hydrogen-bonding effect, which can preserve the EPS structure and biofilm stability.

**Figure 10:**
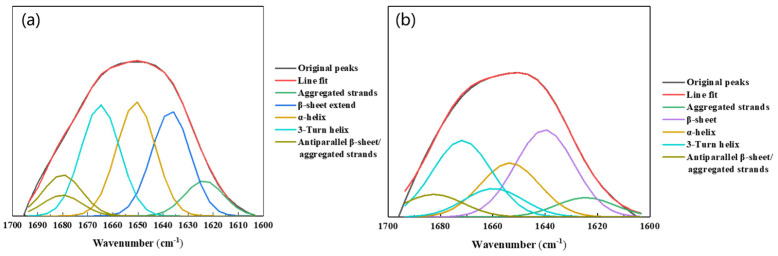
FTIR spectra of the amide I region in the EPS: (**a**) biofilm cells and (**b**) planktonic cells.

The potential effects of biofilm formation on the EPS conformation on metal surfaces were explored to gain insight into the protein structure and amide I region in the EPS of biofilm and planktonic cells. The amide I region (1600–1700 cm^−1^) was deconvoluted using second-order derivative analysis and curve fitting according to the procedure described in [34]. The results showed that the amide I region consisted predominantly of β-sheet, α-helix, 3-Turn helix, and antiparallel β-sheets/aggregated strands, and that the percentage of each assemblage was determined by the ratio of the individual area to the total area (Figure 10 and Table 2). The proteins in the EPS of biofilm cells predominantly showed β-type proteins. The 3-turn helix and antiparallel β-sheet/aggregated chains of proteins in the EPS of biofilms tend to build globular proteins which provide biofilm cellular regulatory functions. Planktonic cells had a higher proportion of α-helix (26.6%) and a lower proportion of antiparallel β-folded/aggregated chains. The α-type protein in the EPS of planktonic cells ensured the individual integrity of free cells. For biofilm cells, a significant proportion of the α-helix may unfold into loops and random structures, and thus the EPS changes its structure due to biofilm formation.

## 4. Discussion

The corrosion behavior of the biofilm of *D. bizertensis* on the surface of Q235 steel, studied using intracellular proteomics and EPS analysis, is discussed and described in detail below.

### 4.1. D. bizertensis Motility Change

Planktonic cells can sense environmental signals released by metal surfaces and actively approach and adhere to metal surfaces by modifying cell-surface biochemical properties and behaviors by regulating gene expression. Fe^2+^ is a cofactor for many oxidoreductase enzymes in SRB, such as cytochromes and ferredoxin [35], and is indispensable for SRB. Some studies reported that Fe^2+^ is an elicitor for the electrophiles *Geobacter metallireducens* and *Shewanella oneidensis,* which use their chemotaxis towards Fe^2+^ to direct the bacteria towards insoluble electron acceptors [36,37]. Chemotaxis is the process by which microorganisms sense and respond to changes in the environment through the regulation of gene expression [38]. Bacterial chemotaxis involves the chemosensory pathway, which is part of the signal transduction system of the binary system (TCS) pathway [39]. In bacteria, the chemotaxis system consists of methyl-accepting chemotactic proteins (MCP), cytoplasmic chemotactic proteins (Che proteins), and flagellar regulation. Chemotaxis depends on the spatial gradient of environmental factors [40]. Bacterial chemoreceptors are very sensitive and can sense extracellular specific ligands at concentrations ranging from μM to nM [41]. In cultures containing Q235 carbon steel, there was found a spatial gradient in Fe^2+^ concentration before Q235 carbon steel was corroded by bacteria [42], and the chemotaxis of SRB bacteria toward Fe^2+^ regulates biofilm formation on the steel surface, as evidenced by proteomics data. Chemotaxis protein CheW (DS_GMQ000927) encoding proteins involved in the regulation of signal transduction systems are up-regulated in biofilm cells.

Chemotaxis-associated proteins regulate bacterial motility by modulating flagellar behavior. Flagellar expression increases when cells sense increased concentrations of elicitors [43]. Proteomics has shown that many flagellar-associated proteins are up-regulated (Figure 4). The increase in flagellar velocity increases bacterial motility, which can allow bacteria to reach the surface of the material faster under the influence of chemotaxis; this facilitates the attachment of bacteria to the surface of Q235.

Proteomics data revealed that Bis-(3′-5′)-cyclic dimeric GMP (c-di-GMP)-related proteins (DS_GM000988 PilZ domain-containing protein, DS_GM003056 GGDEF domain-containing protein) were strongly regulated and up-regulated, 2.01- and 2.24-fold, respectively. Bis-c-di-GMP is a ubiquitous bacterial second messenger involved in the regulation of virulence, cell surface-associated traits, and biofilm formation, stimulating the expression of various adhesions and biofilm-associated exopolysaccharides [44,45]. The components of the c-di-GMP regulatory network include proteins of the GGDEF and EAL structural domains, which determine their cell concentration by mediating the synthesis and degradation of c-di-GMP, respectively. The c-di-GMP is recognized by receptors containing the PilZ structural domain for signal transduction [46]. From this, *D. bizertensis* cells were attracted to form biofilm on Q235 steel through prevalent mechanisms such as chemotaxis and motility. It has been reported that some short proline-rich peptides with cationic and aromatic groups bind with c-di-GMP with nanomolar affinity, thereby blocking the c-di-GMP signaling pathway, which inhibits bacterial attachment and subsequent biofilm formation on metal surfaces [47,48,49].

### 4.2. Protein Secretion and Transport in Cells

The proteins in the EPS play a dominant role in biofilm formation [50]. By KEEG enrichment, the secretion system (map03070) and protein export (map03060) of *D. bizertensis* were significantly up-regulated in biofilm cells, compared to planktonic cells, indicating that the bacteria produce more extracellular proteins. This may account for higher protein concentration in the EPS. It has been reported that the bacterial Sec system is the main pathway responsible for exporting proteins across the cytoplasmic membrane and inserting proteins into the cytoplasmic membrane [51]. The FtsY is considered to be the SRP receptor [52]. The *D. bizertensis* protein contains signaling peptides (DS_GM002752 signal recognition particle-docking protein FtsY) for the ubiquitous Sec secretion pathway, which bring proteins to the targeted extracellular environment. The lipid-anchored protein ApbE was found to be significantly up-regulated in biofilm cells relative to planktonic cells; ApbE (DS_GM000592) lipoproteins are part of the ApbE superfamily and are periplasmic lipoproteins anchored to the inner membrane [53]. Periplasmic ApbE lipoproteins are involved in thiamine biosynthesis [54] and the iron–sulfur cluster metabolism [55].

### 4.3. Survival and Electron Transfer Components of Biofilm Cells

SRB biofilms on Q235 surfaces are usually very dense [56]. As shown in Figure 1a,b, *D. bizertensis* formed dense biofilms on the Q235 surface, and those biofilms may act as an important mass transfer barrier by impeding the diffusion of organic carbon and sulfate to the iron’s surface. Nutrient consumption by organisms in the upper layers of biofilm can lead to starvation of organisms in the lower biofilm layers, which may lead to the adoption of slow growth states in dormant cells [57]. For instance, organic carbon in the native fluid phase may not be utilized by cells beneath the SRB biofilm [13], which may lead to the slowing of the growth of *D. bizertensis* at the bottom of the biofilm. Proteomics data suggest that the ribosomal proteins and proteins related to cellular metabolism such as carbon metabolism, sulfur metabolism, etc., are down-regulated in biofilm cells, because the extent of metabolism depends on the activation step used for substrate metabolism and the terminal electron acceptance conditions. Xu and Gu (2014) reported that Fe0 can also be used as an alternative donor by SRB during energy metabolism when the source of the electron donor is absent or limited [13]. Thus, in the absence of the available carbon source, *D. bizertensis* at the bottom of the biofilm may utilize Q235 as an electron donor to obtain energy for survival, resulting in corrosion on Q235 steel. Electrochemical Reaction (1) and Reaction (2) below illustrate SRB respiration for energy production using elemental iron as the electron donor, while sulfate is the terminal electron acceptor:

4Fe → 4Fe^2+^ + 8e^−^ (*E*° = −447 mV) (R1)

SO_4_^2−^ + 9H^+^ + 8e^−^ → HS^−^ + 4H_2_O (*E*°′ = −217 mV) (R2)

in which *E*°′ stands for the reduction potential (vs. SHE or standard hydrogen electrode) at 25 °C, pH 7 (denoted by the apostrophe), and 1 M solutes (or 1 bar gases) excluding H^+^ in bioelectrochemistry [58]. The cell potential (Δ*E*°′) is +230 mV for the overall reaction coupling Reactions (1) and (2) above. The *E*°′ value for Fe^2+^/Fe (−447 mV) is slightly more negative than that of CO_2_ + acetate/lactate (−430 mV), which means Fe^0^ is slightly more energetic than lactate under the conditions defined for *E*°′. For the redox reaction combining Fe^0^ oxidation and sulfate reduction, the Gibbs free energy change of reaction is ΔG°′ = −178 kJ/mol sulfate (under the conditions defined for *E*°′), which is slightly more negative (i.e., slightly more energy released) than the ΔG°′ = −164 kJ/mol sulfate value for lactate reduction coupled with sulfate reduction, indicating that the corrosion reaction is thermodynamically favorable [13,58].

Increased adhesion of bacteria onto substrate surfaces is a survival strategy under energy limitation. In oligotrophic environments, the increased adhesion of bacteria to the substrate may be due to the adsorbed substrate being more energy-rich than the surrounding environment, creating a favorable nutrient environment for survival around the adsorbed substrate [59,60]. Many microbial strains exhibit increased surface adhesion under energy limitation. For instance, Gram-negative *Escherichia coli* and *Shewanella oneidensis* induce adhesion by increasing the hydrophobicity of their outer cell membrane [61] (Saini et al., 2011), whereas *Vibrio* sp. strain DW1 undergoes reduced cell division and subsequently produces bridging polymers that enhance adhesion to surfaces under starvation conditions [62]. The presence of o-acetylated carbohydrates on lipopolysaccharides is thought to play a key role in cell aggregation and adhesion [63]. Proteomics data revealed that a significant up-regulation of LPS exported ABC transporter periplasmic protein LptC (DS_GM000883).

The energy conservation in SRB is studied using the hydrogen cycle mechanism proposed by Odom and Peck (1981). In this mechanism, protons and electrons from the oxidation of lactate and pyruvate react with cytoplasmic hydrogenase to form hydrogen, which diffuses to the membrane, where it is reoxidized by periplasmic hydrogenase to form a proton gradient. This proton gradient is used for ATP synthesis [64]. This intracellular redox cycle has been extended to include other possible intermediates such as formate and carbon monoxide [65]. CO dehydrogenase is an essential hydrogenase for the production of H_2_ from lactate. Proteomics data from *D. bizertensis* indicated the presence of CO dehydrogenase (DS_GM000468), which was localized towards the cytoplasmic active site. Hydrogen production from CO is determined according to the overall equation CO + H_2_O → CO_2_ + H_2_; the hydrogen produced could then be captured by periplasmic hydrogenases to form a proton gradient [66]. There may be alternative pathways in *D. bizertensis* that lead to pyruvate fermentation of hydrogen or CO production, and the CO produced could then be captured by periplasmic carbon monoxide dehydrogenases for reoxidation. The Rnf complex is a membrane-bound, iron–sulfur, and flavin electron transport complex encoded by the genomes of many bacteria and some archaea [67,68]. NAD was regarded as the most electronegative electron donor in classical bioenergetics, but in recent years ferredoxin has been a widely used electron carrier in anaerobes [69,70]. Ferredoxins are used as primary electron acceptors for hydrogenases, formate dehydrogenases, and CO dehydrogenases [71,72,73]. In addition, some anaerobic bacteria couple the exergonic reduction of fermentation intermediates to the endergonic reduction of ferredoxin with NADH as a reductant in a process called “electron bifurcation” [69,74]. Ferredoxin: NAD oxidoreductase (Fno) activity is directly coupled to Na translocation, whereas with Na translocation, Fno activity is catalyzed by the Rnf complex. The bound Rnf complex mediates electron transfer between NADH and Fd. *D. bizertensis* encodes for two Rnf complexes (DS_GM000588 and DS_GM000587), both of which were expressed under the tested conditions (Table S1) and are believed to be involved in energy conservation (H/Na-based bioenergetics) in *D. bizertensis* [75,76]. The presence of the Rnf complex suggested that it might have played a key role in the energy conservation strategies of *D. bizertensis*.

It has been found that [FeFe] Hases catalyze the reduction of Fd and NADH as electron donors for H_2_ production and that the ion-translocating complex Rnf, together with Coo Hases, provides a coupling site for Fd-related processes such as electron bifurcation [76,77]. Proteomics analysis revealed that [FeFe] hydrogenase (DS_GM002404) was up-regulated in biofilm cells. [FeFe] Hases provided further evidence that, under sulfate restriction in biofilm bottom cells, SRB operates a dual pathway in response to sulfate availability, and [FeFe] Hases catalyzes the reduction of Fd and NADH to release electrons for energy supply [77,78,79].

### 4.4. EPS Analysis

The fluorescence staining and quantitative analysis of EPS (Figure 1d–f and Figure 8) showed that the main composition of EPS from the strain *D. bizertensis* was proteins, followed by polysaccharides. This was consistent with the compositions of EPS in many other reports [80,81]. The role of extracellular proteins in biofilm formation by *D. bizertensis* on Q235 surfaces was studied by analyzing the secondary structure of proteins in EPS (Figure 10 and Table 2). Microorganisms aggregate and form biofilms when protein secondary structures like β-sheets, α-helix, 3-Turn helix, and aggregated strands are high in EPS. However, when protein secondary structures like Antiparallel β-sheet/aggregated strands are high in biofilm EPS, the aggregation and biofilm formation ability is inhibited [82]. As shown in Table 2, the protein secondary structures like β-sheet, α-helix, 3-turn helix, and aggregated strands were present in 90% of the EPS of *D. bizertensis* biofilm cells, which may have played a crucial role in SRB biofilm formation. C=O groups in EPS play a dominant role in Fe–EPS conjugation binding, causing the transformation of protein secondary structure (especially β-sheet and α-helix), through the correlation between Fe and carbonyl oxygen/amide hydrogen binding, which disrupts the secondary structure represented by α-helix and forms β-sheet. α-helix is retained through the hydrogen bonding connection formed between the amide hydrogen and the carbonyl oxygen of the peptide bond [83]. The results of XPS showed that the percentage of C=O/O-C=O in biofilm EPS was about 71.9%, while the percentage of C=O/O-C=O in the planktonic cells of EPS was 49.3%, indicating the EPS in the biofilm contained a stronger binding ability relative to Fe than did the EPS of the planktonic cells, which allows the biofilm to be attached stably on the surface of Q235. An increase in the proportion of β-sheets in EPS increases the chelation of the organic matter with metal ions [84]. The percentages of α-helix in EPS of planktonic and biofilm cells were 26.6% and 18.3%, respectively (Table 2). In addition, the percentage of β-sheet in EPS of biofilm cells was 29.5% whereas no β-sheet was observed in planktonic EPS. This suggests that Fe disrupts the α-helix in the biofilm EPS and converts it to β-sheet. Further, the EPS chelates Fe^2+^ to promote anodic dissolution of Q235 [85], and EPS-bound metal ions can act as electron “shuttles” and create new redox pathways in the biofilm/metal system, such as direct electron transfer from metals (e.g., Fe) or minerals (e.g., FeS) for direct electron transfer to ensure energy supply to biofilm cells.

## 5. Conclusions

In this paper, we explored the cause of Q235 corrosion by *D. bizertensis* biofilm through intracellular proteomics and EPS compositional analysis. Proteomics analysis showed that biofilm formation enhanced cellular motility and chemotaxis, upregulated the flagellum-associated proteins, and increased intercellular aggregation and bacterial adhesion through the c-di-GMP second messenger molecule, suggesting that Q235 is attractive to SRB. After biofilm formation, the metal substrate is energy-rich due to nutrient consumption by organisms in the upper layers of biofilm, and *D. bizertensis* utilizes Q235 as an electron donor to maintain itself at a minimum energy level. Further, the up-regulation of the related membrane proteins Rnf complex, Coo hydrogenase, and iron hydrogenase in the biofilm cells was over-expressed. The compositional analysis of EPS showed that proteins are the main components in EPS, and their secondary structure was mainly β-sheet. Further, the increases of β-sheet in the EPS increase the chelation with Fe^2+^ ions and accelerate the corrosion of the metal. The findings revealed that higher content levels of β-sheet, α-helix, 3-Turned helix, and aggregated strands in EPS lead to increased aggregation and biofilm formation by *D. bizertensis*. In the future, coatings can be utilized to reduce the adhesion of SRB, and biocides can be utilized to destroy the structure of the EPS of SRB, thereby, reducing the corrosive effects of SRB on metallic materials.

## Figures and Tables

**Table 1:** Characteristics, binding energy, and possible structure of EPS from planktonic and biofilm cells analyzed using XPS. Binding energies were referenced to C 1s and O 1s.

Cells	Element	Binding Energy (eV)	Relatives Content	Proposed Components
**Plankton**	**C 1s**	284.4	23.50%	Aromatic carbon
		284.8	14.30%	C-(C,H)
		285.1	25.30%	C-N
		286.1	23.90%	C-(O,N)
		288.1	13%	N-C=O
	**O 1s**	531.3	50.70%	C=O/O-C=O
		532.3	49.30%	C-O-H/C-O-C
		533	0%	OH-C=O
**Biofilm**	**C 1s**	284.4	23%	Aromatic carbon
		284.8	13.10%	C-(C,H)
		285.1	21.80%	C-N
		286.1	25.10%	C-(O,N)
		288.1	17.10%	N-C=O
	**O 1s**	531.3	71.90%	C=O/O-C=O
		532.3	11.90%	C-O-H/C-O-C
		533	12.90%	OH-C=O
		535.8	3.30%	H2O

**Table 2:** Wavenumber and secondary structure of proteins in the EPS of planktonic and biofilm cells (%).

Secondary Structures	Wavenumber (cm^−1^)	Planktonic	Biofilm
Unknown	1605–1600	0	2.3
Aggregated strands	1625–1610	8.3	6.6
β-sheet extend	1640–1630	24.6	0
β-sheet	1645–1640	0	29.5
α-helix	1657–1648	26.6	18.3
3-Turn helix	1676–1659	26	35.6
Antiparallel β-sheet/aggregated strands	1695–1680	14.5	7.7

## Data Availability

The raw data supporting the conclusions of this article will be made available by the authors on request.

## Supplementary Materials

The following supporting information can be downloaded at: https://www.mdpi.com/article/10.3390/ma17205060/s1. Figure S1: SEM and LIVE/DEAD staining of *D. bizertensis* after EPS extraction; Figure S2: Subcellular location of differentially expressed proteins; Table S1: Description and changed folds of all up-regulated and down-regulated differentially expressed proteins (DEPs) in cells of biofilm and planktonic; Table S2: Description and changed folds of some up-regulated and down-regulated differentially expressed proteins (DEPs) in cells of biofilm and planktonic; Table S3: Functional category distribution of differentially expressed proteins by GO in cells of biofilm and planktonic; Table S4: KEGG analysis of the up-regulated DEPs between biofilm cells on the Q235 surface and planktonic cells; Table S5: KEGG analysis of the down-regulated DEPs between biofilm cells on the Q235 surface and planktonic cells; Table S6: Peptide fraction separation liquid chromatography elution gradient table; Table S7: Liquid chromatography elution gradient table. References [86,87,88,89,90,91,92] are cited in the supplementary materials.
