# Supplementary material for: Proteomics and EPS Compositional Analysis Reveals Desulfovibrio bisertensis SY-1 Induced Corrosion on Q235 Steel by Biofilm Formation
Source: Materials (Basel). 2024 Oct 17;17(20):5060. doi: 10.3390/ma17205060 (PMC11509735; doi:10.3390/ma17205060)
Supplement: Supplementary file 1 [file materials-17-05060-s001.zip › materials-3226793-supplementary.pdf]

Proteomics and EPS Compositional Analysis Reveals *Desulfovibrio bisertensis* SY-1 Induced Corrosion on Q235 Steel by Biofilm Formation

Yanan Wang [1,2,3], Ruiyong Zhang [1,3,*,†], Krishnamurthy Mathivanan [1,3], Yimeng Zhang [1,3], Luhua Yang [1,3], Fang Guan [1,3,†] and Jizhou Duan [1,3,*,†]

1 Key Laboratory of Advanced Marine Materials, Key Laboratory of Marine Environmental Corrosion and Bio-Fouling, Institute of Oceanology, Chinese Academy of Sciences, Qingdao 266071, China
2 University of Chinese Academy of Sciences, Beijing 100049, China
3 Center for Ocean Mega-Science, Chinese Academy of Sciences, Qingdao 266071, China
* Correspondence: ruiyong.zhang@qdio.ac.cn (R.Z.); duanjz@qdio.ac.cn (J.D.); Tel.: +86-532-82898851 (R.Z. & J.D.)
† Current address: Guangxi Key Laboratory of Marine Environmental Science, Institute of Marine Corrosion Protection, Guangxi Academy of Sciences, Nanning 530007, China.

Materials and Methods

Proteome analysis

1.1 *D. bizertensis* collection

Bacteria were collected by centrifugation and rinsed quickly with pre-cooled PBS 2-3 times to avoid contamination of bacteria by medium proteins. After each wash, the material was centrifuged at 4°C, 5000g for 5min to discard the supernatant completely, and the bacterial body was collected in 1.5mL imported centrifuge tubes (cryopreservation tubes), quick-frozen in liquid nitrogen, and stored in the refrigerator at -80°C.

1.2 Total Protein Extraction

(Kachuk et al. 2015, Marx et al. 2016, Niu et al. 2018, Wiśniewski et al. 2009, Wu et al. 2014)

The sample was transferred to a 1.5ml centrifuge tube and lysed with SDT lysis buffer (4% SDS, 10 mM DTT(DL-Dithiothreitol), and 100 mM TEAB (triethylammonium bicarbonate)); this was followed by 5 min of ultrasonication on ice. The lysate was centrifuged at 12000 g for 15 min at 4°C and the supernatant was reduced with 10 mM DTT for 1 h at 56°C and subsequently alkylated with sufficient iodoacetamide for 1 h at room temperature in the dark. Then, the samples were completely mixed with 4 times volume of precooled acetone by vortexing and incubated at -20°C for at least 2 h. Samples were then centrifuged at 12000 g for 15 min at 4°C and the precipitation was collected. After washing with 1mL cold acetone, the pellet was dissolved by dissolution buffer (8 M Urea, 100 mM TEAB, pH 8.5).

2. Protein Quality Test

BSA standard protein solution was prepared according to the instructions of the Bradford protein quantitative kit, with gradient concentrations ranging from 0 to 0.5 g/L. BSA standard protein solutions and sample solutions with different dilution multiples were added into the wells of a 96-well plate to fill up the volume to 20 μL, respectively. Each gradient was repeated three times. A quantum of 180 μL G250 dye solution was quickly added to the plate, and it was placed at room temperature for 5 minutes; the absorbance at 595 nm was then detected. The standard curve was drawn with the absorbance of standard protein solution, and the protein concentration of the sample was calculated. A quantity of 20 μg of the protein sample was loaded to 12% SDS-PAGE gel electrophoresis, wherein the concentrated gel was assayed at 80 V for 20 min, and the separation gel was assayed at 120 V for 90 min. The gel was stained by Coomassie brilliant blue R-250 and decolored until the bands were visualized clearly.

3. TMT Labeling of Peptides

(Zhang et al. 2016)

Each protein sample was taken, and the volume was increased to 100 μL with DB dissolution buffer (8 M Urea, 100 mM TEAB, pH 8.5). Trypsin and 100 mM TEAB buffer were added, and the sample was mixed and digested at 37 °C for 4h. Then, trypsin and $CaCl_2$ were added, and the sample was digested overnight. Formic acid was mixed with the digested sample, the pH was adjusted to under 3, and the sample was centrifuged at 12000 g for 5 min at room temperature. The supernatant was slowly loaded to the C18 desalting column, washed with washing buffer (0.1% formic acid and 3% acetonitrile) 3 times, and then eluted by some elution buffer (0.1% formic acid and 70% acetonitrile). The eluents of each sample were collected and lyophilized. In total, 100 μL of 0.1 M TEAB buffer was added to reconstitute the samples, and 41 μL of acetonitrile-dissolved TMT labeling reagent was added as well. The sample was mixed by shaking for 2 h at room temperature. After this, the reaction was stopped by adding 8% ammonia. All labeling samples were mixed with equal volumes, desalted, and lyophilized.

Note: For multiple labelled groups, a common reference was created by pooling an equal quantity of each sample.

4. Separation of fractions

Mobile phases A (2% acetonitrile, adjusted pH to 10.0 using ammonium hydroxide) and B (98%

acetonitrile) were used to develop a gradient elution. The lyophilized powder was dissolved in solution A and centrifuged at 12,000 g for 10 min at room temperature. The sample was then fractionated using a C18 column (Waters BEH C18, 4.6×250 mm, 5 μm) on a Rigol L3000 HPLC system; the column temperature was set at 45°C. The detail of the elution gradient is shown in Table S1.

The eluates were monitored at UV 214 nm, collected in a tube for one minute, and finally combined into 10 fractions. All fractions were dried under vacuum, and then reconstituted in 0.1% (v/v) formic acid (FA) in water.

Table S6: Peptide fraction separation liquid chromatography elution gradient table.

Time (min)	Flow Rate (nL/min)	Mobile Phase A (%)	Mobile Phase B (%)
0	1	97	6
10	1	95	5
30	1	80	20
48	1	60	40
50	1	50	50
53	1	30	70
54	1	0	100

5. LC-MS/MS Analysis

For transition library construction, shotgun proteomics analyses were performed using an EASY-nLCTM 1200 UHPLC system (Thermo Fisher) coupled with a Q Exactive TM HF-X mass spectrometer (Thermo Fisher) operating in the data-dependent acquisition (DDA) mode. The 1 μg sample was injected into a home-made C18 Nano-Trap column (4.5 cm×75 μm, 3 μm). Peptides were separated in a home-made analytical column (15 cm×150 μm, 1.9 μm), using a linear gradient elution as listed in Table S2. The separated peptides were analyzed by a Q Exactive TM HF-X mass spectrometer (Thermo Fisher), with an ion source of Nanospray Flex™（ESI）, spray voltage of 2.1 kV, and ion transport capillary temperature of 320°C. Full scans ranged from m/z 350 to 1500, with resolution of 60000 (at m/z 200). The automatic gain control (AGC) target value was 3×10^6 and the maximum ion injection time was 20 ms. The top 40 precursors of the highest abundance in

the full scan were selected and fragmented by higher energy collisional dissociation (HCD) and analyzed in MS/MS, for which the resolution was 30000 (at m/z 200) for 6 plex, the automatic gain control (AGC) target value was 5×104, the maximum ion injection time was 54 ms, the normalized collision energy was set as 32%, the intensity threshold was 1.2×10^5, and the dynamic exclusion parameter was 20 s. The raw data from the MS detection was saved as a file with the filetype ".raw"

Table S7: Liquid chromatography elution gradient table.

Time (min)	Flow Rate (nL/min)	Mobile Phase A (%)	Mobile Phase B (%)
0	600	94	6
2	600	85	15
48	600	60	40
50	600	50	50
51	600	45	55
60	600	0	100

6. Data analysis

6.1 The identification and quantitation of proteins

The spectra resulting from each run were searched separately against the 1234349-Desulfovibrio singaporenus. fasta(3406 sequences) database, using the search engine Proteome Discoverer 2.4 (PD 2.4, Thermo). The search parameters were set as follows: mass tolerance for precursor ion was 10 ppm and mass tolerance for product ion was 0.02 Da. Carbamidomethyl was specified as fixed modifications, and Oxidation of methionine (M) and TMT plex were specified as dynamic modification. Acetylation, TMT plex, Met-loss, and Met-loss+Acetyl were specified as N-Terminal modifications in PD 2.4. A maximum of 2 missed cleavage sites were allowed.

In order to improve the quality of the results of the analysis, the software PD 2.4 was used to further filter the retrieved results: Peptide Spectrum Matches (PSMs) with a credibility of more than 99% were termed identified PSMs. The identified protein contained at least 1 unique peptide. The identified PSMs and protein were retained and tested with FDR to levels of no more than 1.0%. The protein quantitation results were statistically analyzed by means of a T-test. The proteins for which the quantitation results were significantly different between the experimental and control groups (p

< 0.05 and FC > 1.5 or FC < 0.67 [FC, fold change]) were defined as differentially expressed proteins (DEP).

6.2 The functional analysis of protein and DEP

Gene Ontology (GO) and InterPro (IPR) functional analyses were conducted using the interproscan program against the non-redundant protein database (including Pfam, PRINTS, ProDom, SMART, ProSite, and PANTHER) (Kachuk et al. 2015), and the databases associated with COG (Clusters of Orthologous Groups) and KEGG (Kyoto Encyclopedia of Genes and Genomes) were used to analyze the protein families and pathways. DEPs were used for volcanic map analysis, cluster heat map analysis, and enrichment analysis of GO, IPR, and KEGG (Wiśniewski et al. 2009). The probable protein–protein interactions were predicted using the STRING-db server (Gillette et al. 2020) (http://string.embl.de/)

Supplemental Figures

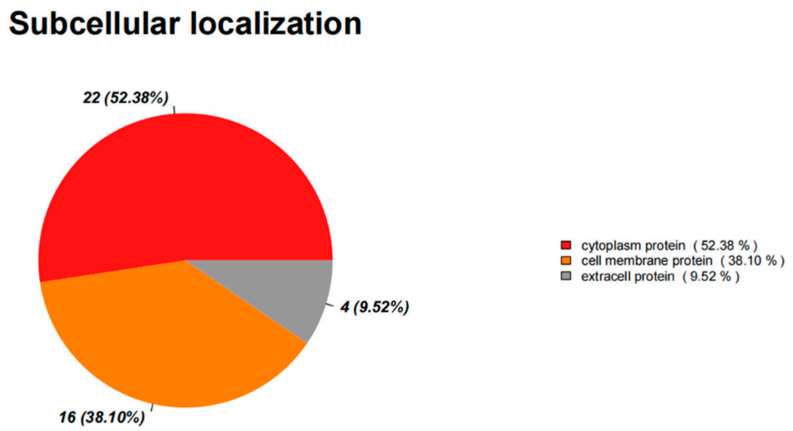

Figure S1. Subcellular location of differentially expressed proteins.

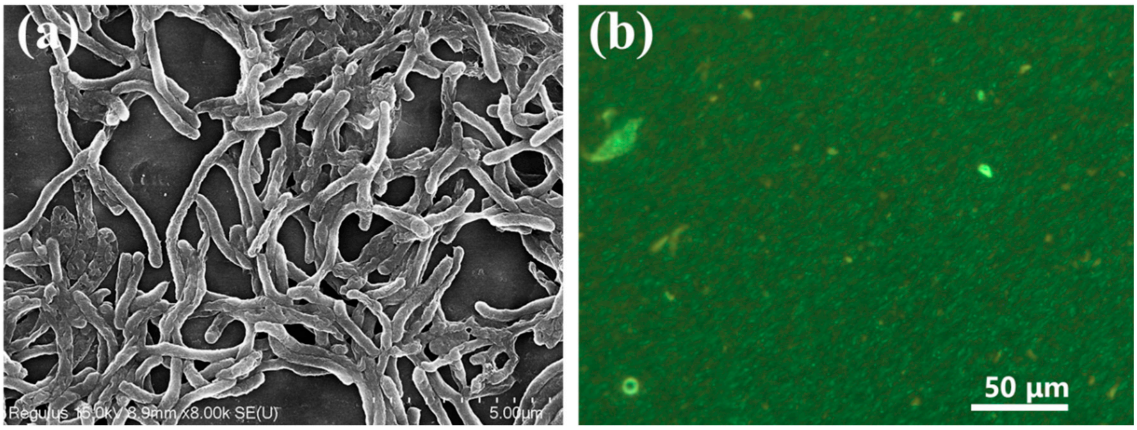

Figure S2. SEM and LIVE/DEAD staining of *D. bizertensis* after EPS extraction.

References

Gillette, M.A., Satpathy, S., Cao, S., Dhanasekaran, S.M., Vasaikar, S.V., Krug, K., Petralia, F., Li, Y., Liang, W.-W. and Reva, B. (2020) Proteogenomic characterization reveals therapeutic vulnerabilities in lung adenocarcinoma. Cell 182(1), 200-225. e235.

Kachuk, C., Stephen, K. and Doucette, A. (2015) Comparison of sodium dodecyl sulfate depletion techniques for proteome analysis by mass spectrometry. Journal of chromatography A 1418, 158-166.

Marx, H., Minogue, C.E., Jayaraman, D., Richards, A.L., Kwiecien, N.W., Siahpirani, A.F., Rajasekar, S., Maeda, J., Garcia, K. and Del Valle-Echevarria, A.R. (2016) A proteomic atlas of the legume Medicago truncatula and its nitrogen-fixing endosymbiont Sinorhizobium meliloti. Nature biotechnology 34(11), 1198-1205.

Niu, L., Zhang, H., Wu, Z., Wang, Y., Liu, H., Wu, X. and Wang, W. (2018) Modified TCA/acetone precipitation of plant proteins for proteomic analysis. PloS one 13(12), e0202238.

Wiśniewski, J.R., Zougman, A., Nagaraj, N. and Mann, M. (2009) Universal sample preparation method for proteome analysis. Nature methods 6(5), 359-362.

Wu, X., Xiong, E., Wang, W., Scali, M. and Cresti, M. (2014) Universal sample preparation method integrating trichloroacetic acid/acetone precipitation with phenol extraction for crop proteomic analysis. Nature protocols 9(2), 362-374.

Zhang, H., Liu, T., Zhang, Z., Payne, S.H., Zhang, B., McDermott, J.E., Zhou, J.-Y., Petyuk, V.A., Chen, L. and Ray, D. (2016) Integrated proteogenomic characterization of human high-grade serous ovarian cancer. Cell 166(3), 755-765.
